# Effect of nitrogen (N) deposition on soil-N processes: a holistic approach

**DOI:** 10.1038/s41598-020-67368-w

**Published:** 2020-06-26

**Authors:** Preeti Verma, R. Sagar

**Affiliations:** Department of Botany, Institute of Science, Banaras Hindu University, Varanasi, 221005 India

**Keywords:** Ecology, Ecology

## Abstract

Nitrogen (N) deposition is a serious environmental issue for soil fertility and human wellbeing. Studies on various terrestrial ecosystems yielded fragmented information on soil-N status (microbial biomass- and mineral-N) and dynamics (N-mineralization and -leaching) whereas the holistic view on this issue is relatively unknown. A complete understanding of soil-N status and dynamics in response to N deposition is essential for sustainable management of ecosystem structure and function as needed for human wellbeing. Therefore, we conducted an experiment in the N-limited tropical grassland to explore the question whether N-deposition weakens the soil-N status and dynamics; if yes, then what could be the optimum amount of deposited N and the related controlling mechanism? We undertook a 3-year (2013–2016) experimental N fertilization (control, 30, 60, 90, 120, and 150 kg N ha^−1^ year^−1^) study (using urea as a source of N deposition). The data from a total of 72, 1 × 1 m plots (six treatments with 12 replicates) were collected and properly analysed with statistical software. N deposition caused significant differences in the parameters of soil-N status and dynamics. The responses of microbial biomass-N, N-mineralization, and mineral-N to the N deposition were quadratic (maximum values were in N_90_) whereas N-leaching showed a linear response. Compared to control, N deposition (30–150 kg N) consistently enhanced (29–96%) leaching of N. As a mechanism, acidification induced aluminium toxicity, carbon to nitrogen ratio and litter decomposition governed the soil-N status and dynamics. N deposition over and above 90 kg ha^−1^ year^−1^ resulted in a negative feedback to soil N transformation and availability. Hence, N deposition below 90 kg ha^−1^ year^−1^ could be a limit for the sustainable functioning of the tropical or similar grasslands.

## Introduction

Globally, N deposition has been identified as a major threat to the functioning of the sensitive ecosystems^1,2^. Fossil fuel combustion, biomass burning, changes in land use pattern and use of N-fertilizer have been identified as major contributors of atmospheric-N depositions^1,2^.These N deposition sources doubled the global N cycle over the last century^3^. Studies have suggested that in 1,860 the reactive-N deposition for terrestrial ecosystems was 15.88 Tg year^−1^ and at the beginning of the 1990s it was 63.5 Tg year^−1^ which is four times higher than that in 1860^4^. According to Galloway et al.^5^ and Zhou et al.^6^, recently, the global ecosystems are receiving a very high rate of N deposition, often > 100 kg N ha^−1^ year^−1^ and it may reach up to 125.2 Tg year^−1^ by 2050^4,7^. The deposition is expected to increase by a factor of 2.5 over the next century^8^. In the Asian region, the predicted reactive-N deposition by 2030 would be more than 1.5 times higher (from 67.7 to 105.3  Tg year^−1^) than that of 2000^9^ and by 2020 it is likely to exceed the combined emissions of North America and Europe^10^. On the other hand, the global N fertilizer uses in 1960 and 2000 were 3.5 and 87 million metric tons (MT) and by 2050, it would be around 249 million MT^1^. These estimates indicated that between 1960 and 2000, annual N fertilizer use was 2.09 million MT and by 2050 it would increase by 2.73 million MT annually. To feed the hungry world (through massive food and agriculture production) has been identified as a major reason for tremendous hike in the global N fertilizer use and emission. These studies reflected synchronization of atmospheric-N deposition and N fertilization^1,2,11–13^ (henceforth, N fertilization is referred to as N deposition).

The N deposition and its associated processes responsible for changes in structure and functioning of the ecosystems constitute a big challenge to the mankind, and in future, the situation would be horrific^11–13^. Studies from various terrestrial ecosystems showed disturbances in normal soil-N status (NH_4_^+^-N, NO_3_^−^-N, total mineral-N, microbial biomass nitrogen; MBN) and dynamics (ammonification, nitrification, N mineralization and rate of soil-N leaching) owing to atmospheric N depositions^14–19^. These perturbed N status and dynamics have changed plant composition, diversity^20^, productivity and carbon storage capacity of the concerned ecosystems^5,21,22^. Due to N deposition induced changes in the normal N cycling^23^, further increasing rate of N deposition is assumed to be one of the main modulators of the global climate change^19,24,25^ which is seriously threatening the human well-beings^5,26–28^.

Among the soil N pools, MBN has been observed as an indicator of soil fertility and normal N cycling^29,30^. Reports suggested decreasing^30^, increasing^31^ and no^32^ effect of N deposition on the MBN. Thus the responses of MBN to N depositions are inconclusive and need more studies to conclude. Therefore, the feedbacks of MBN to the atmospheric-N depositions from diverse ecosystems need urgent study for predicting its impact on the soil health and global N cycling^33^.

Soil-N mineralization is a fundamental step of soil-N transformation^33^, governing the status of soil fertility^34^ and fate of NO_3_^−^ leaching^35^. The research on the response of N-mineralization to N-deposition dates back to the early 1970s, since then the results are fragmentry and indecisive; viz: increased^36–38^, decreased^30,39^ and no pattern^40–42^. Hence; similar to MBN, the assessment of N deposition effect on soil-N mineralization from different ecosystems are imperative^43^.

The excess N deposition leads to high N availability and causes N saturation^44^, although N deposition may increase soil N-mineralization in the N-limited ecosystems, and decrease the MBN as well as N retention capacity of the soil^45^. The excess N goes beyond the availability of plants and microbes and is lost through leaching^46,47^ or gaseous emissions^44^. Therefore, determination of N deposition threshold of an ecosystem prior to occurrence of negative impacts on its soil-N pools and processes is essential. Determining the N threshold will be helpful to the policymakers for curbing the N loss via gaseous and soil-N leaching^23,45^.

Temperate and sub-tropical studies have improved our understanding of the fate of soil-N leaching in response to N deposition. These studies suggested increased N-leaching on account of increased N deposition^48^. This pattern has been argued because of increased N availability and decreased N retention capacity of the soil^49^. Similar to other parameters of soil-N pools and processes, our understanding on the response of N leaching to the N deposition from the tropical grasslands is not much known. It is expected that the response of N leaching in tropical ecosystems may differ from that of temperate and sub-tropical ecosystems because tropical ecosystems are not limited to N-saturation^50^ and are characterised by marked seasonality^21,22,29^. Added to it, the response of inorganic N leaching to the N depositions in natural ecosystem is thought to be complex and non-linear, due to differences in climate, vegetation and soil attributes^49^. Hence, from the N management view point and prediction of global N-leaching response to the N deposition, information from tropical ecosystems could be valuable^50^.

The N deposition and its consequences on soil-N status, dynamics and leaching from agroecosystems, grasslands and forests of temperate, tropical and sub-tropical ecosystems are fragmentary^4,7,23,51,52^. Unfortunately these studies lack the holistic response of soil-N status and dynamics to N deposition^53^. On one hand, the tropics are major sources and sink of atmospheric N deposition^4^ compared to other regions^50^ because of tremendous human pressure on per capita utilization of food and energy^7^. On the other hand, these regions are losing their biodiversity at an alarming rate due to high biotic pressure^54^ and N depositions^23,55^. For example, most tropical grasslands are being converted into shrub, semi-desert and desert like ecosystems which are progressively species poor^56^. As, N often limits the growth and survival of plants in majority of ecosystems^23,55^, above situations together with seasonality-governed functioning of the tropical soils^21,22,29^ prompted us to understand the complete fate of soil-N status and dynamics against the N deposition from N-limited tropical grasslands which are experiencing very high rates of anthropogenic pressure and N deposition.

It has been reported that the soil base and non-base cations, soil acidification and N deposition are directly linked^57^; therefore, analysis of base and non-base cations could be useful in understanding the mechanism of soil-N pool and processes in response to the atmospheric N deposition. The out-come of this study could be helpful in determining the optimum amount of N required for the sustainability of the tropical grassland. Also, the current study could be valuable in global generalization of soil-N status and dynamics in view of the increasing levels of N deposition because until recently no study has considered holistic approach for understanding the effects of N deposition on the soil-N status and dynamics. However, studies on individual responses of soil-N pools and processes to the N deposition are frequently available^19,31,32,48,58^.

Looking into the above accounts; we have set a hypothesis that N depositions weaken the soil-N status and dynamics. If the hypothesis is supported, then what could be the optimum amount of deposited N and what could be the controlling mechanism for soil-N status and dynamics for sustainability of tropical grasslands. Specifically, the objectives of the present study were (i) to determine the responses of MBN, rates of N-mineralization, available soil-N and leaching of mineral-N to the N deposition, (ii) to understand the mechanism governing the soil-N pool and processes in relation to the N deposition from a three-year external N-manipulative experiment conducted in a tropical grassland.

## Materials and methods

### Study location

**Figure 1:**
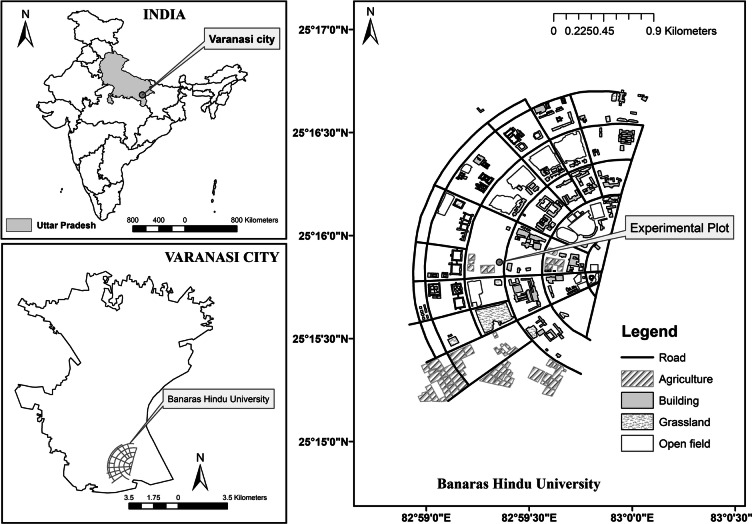
Location of the 3-year of experimental nitrogen (N) inputs plots in Tropical grassland on the campus of Banaras Hindu University, Varanasi, India. Map was created by Dr. P. Verma (co-author of the ms) using ArC Map 10.4 (ESRI, Redland, CA, USA) software and https://desktop.arcgis.com/en/arcmap/.

The study was conducted on 72 plots on the campus of the Banaras Hindu University (24° 18′ N and 83° 03′ E and 76 m m.a.s.l. altitude), during July 2013 to June 2016 in the Indo Gangetic Basin of eastern Uttar Pradesh located in Varanasi district of India (Fig. 1). The climate of the study area is tropical monsoonal with three different seasons; a cold winter (November–February), a hot summer (April–June) and a warm rainy season (July–September). October and March are transitional periods between rainy and winter, and between winter and summer seasons, respectively^59^. During the study period, mean maximum temperature was 31 °C while mean minimum temperature was 20 °C and the mean annual precipitation was 969 mm. The soil is categorised as Banaras Type III^60^, alluvial, well-drained, pale brown, silty loam, inceptisol^61^ and moderately fertile being low in available N and medium in available phosphorus and potassium^62^ with neutral to alkaline soil pH^63^.

The campus of the Banaras Hindu University is spread in ≈ 520 ha land area having a luxuriant growth of natural flora. *Azadiracta indica*, *Dalbergia sissoo*, *Madhuca longifolia*, *Mangifera indica*, *Sterculia alata*, *Tamarindus indica*, *Tectona grandis*, *Zizyphys glaberrima*, etc. are locally top canopy dominating species while *Alysicarpus monilifer*, *Cynodon dactylon*, *Cyperus compressus*, *Desmodium gangeticum*, *Dichanthium annulatum*, *Evolvulus nummularius*, *Imperata cylindrica*, *Malvastrum coromandelianum*, *Oplismenus burmannii*, *Sida acuta* are the locally dominating species of ground vegetation^21,56^.

### Experimental design

A 20 × 20 m open area having natural herbaceous vegetation and substantially away from the buildings was selected in the horticulture premise of the University. Within this; 72, 1 × 1 m experimental plots (plot size determined by species-area curve), arranged in 6 parallel rows (12, 1 × 1 m plots in each row) were established. Surface-to-belowground boundary of each 1 × 1 m plot was permanently demarcated by using bricks and cement (10 cm wide and 50 cm depth). A 1 m distance between two 1 × 1 m plots was kept as buffer zone to protect against boundary effects due to the migration of N out of the sampling areas. Six treatments of N, each replicated twelve times, were randomly established on the basis of lottery method: control (without N), N_30_ (30 kg N ha^−1^ year^−1^), N_60_ (60 kg N ha^−1^ year^−1^), N_90_ (90 kg N ha^−1^ year^−1^), N_120_ (120 kg N ha^−1^ year^−1^) and N_150_ (150 kg N ha^−1^ year^−1^)^64^.

Before the N treatment to the plots; soil bulk density, soil-porosity, -water holding capacity, -sand, -silt and -clay contents for the experimental plots were analysed. Across the plots; values of soil bulk density (g cm^−3^), percentage soil-porosity, -water holding capacity, -sand, -silt and -clay contents varied from 1.23–1.26, 52–54, 48–49, 8–9, 77–79 and 12–13, respectively. Statistically; none of these soil variables varied due to designated N-levels. Thus, before the initiation of the experiment, the selected plots were homogenous in soil characteristics.

### N-inputs

Similar to other studies^65,66^, urea fertilizer was used as a source of N deposition because it has high (46%) N content, zero phosphorus and potassium and is comparatively inexpensive, stable, and easy to handle and it alone contributed more than 50% of the global atmospheric-N deposition^2^. The urea was applied in the evening in each month to avoid the N loss due to volatization^21,64^. For calculating the monthly doses, the total annual N dose of each N level was equally divided into 12 applications. We deliberately used a wide range of N-level with a maximum of 150 kg N ha^−1^ year^−1^ to understand the measureable responses of soil-N attributes and threshold tolerance of soil N to the N deposition within the tropical grassland.

### Sampling and analyses

The soils were sampled at two depths (0–10 cm and 90–100 cm depths from the soil surface). For each N-level, three soil samples (0 to 10 cm depth) were collected from each 1 × 1 m plot, for each month of the year starting from 2013 to 2016 by using a 5 cm-diameter corer. For each soil depth, the three soil samples collected from each 1 × 1 m plot were combined to form a composite soil sample for each plot. These composite soil samples of 0–10 cm depth were gently homogenized. Carefully, large roots, woods, litters and all fine roots were removed from the composite soil samples.

One part of soil sample was air dried, sieved through 2 mm mesh screen and analyzed soil-pH, total organic-carbon (C), total soil-N (TN), ammonium-N (NH_4_^+^-N) and nitrate–N (NO_3_^−^-N). Soils of 0–10 cm depth were used for the analysis of soil N mineralization, microbial biomass carbon; MBC and MBN. To understand the leaching; the soils of 90–100 cm depth were collected by inserting a 100 cm long metallic corer into soil by avoiding the root injury. These soil samples were used for the estimation of NH_4_^+^-N and NO_3_^−^-N^67^. Total soil-N (TN) was determined by micro-Kjeldahl digestion method^68^. NH_4_^+^-N was extracted by 2 M KCl and analyzed by using the phenate method^69^. The concentration of NO_3_^−^-N was analyzed by the phenol disulphonic acid method after extraction by CaSO_4_^68^.

For the analysis of litter decomposition, nylon net litter bag (10 × 10 cm) technique^70^ was adopted. In this analysis, 100 g of air-dried mixed leaf litters of grasses and forbs were used. The mesh size of the litter bags was 1 mm which easily allows the movement and activity of soil microorganisms. Litter decomposition was determined by computing the decay constant (*k*). The negative exponential decay (*k* = − ln (*X*_t_/*X*_0_)/*T*) model was used to compute the *k*^71–73^. In the equation; *X*_0_ is the initial dry weight, *X*_*t*_ the dry weight remaining at the end of the investigation time *T* (1 month).

The in situ buried bag technique was adopted for N-mineralization. Before incubation, the NH_4_^+^-N and NO_3_^−^-N concentrations were determined for zero-month sampling. Using a large sealed polythene bag, a portion of fresh soil sample (200 g) was incubated in soil at a depth of 0–10 cm on the same microsite from which the samples had been collected for the analysis of NH_4_^+^-N and NO_3_^−^-N. After one month of field incubation, the incubated bags were collected for the analysis of NH_4_^+^-N and NO_3_^−^-N. Again, a portion of fresh soil sample (200 g) was incubated on the same microsite from which the samples had been collected for the analyses of NH_4_^+^-N and NO_3_^−^-N and after one month of incubation the incubated samples were re-collected and NH_4_^+^-N and NO_3_^−^-N were re-analyzed. These analyses were repeated for each month of the entire experimental period after an interval of 30 days from 1st July 2013 to 24th June 2016. The increase in the concentrations of NH_4_^+^-N and NO_3_^−^-N after field incubation is referred to as ammonification and nitrification, respectively and the increase in the amount of NH_4_^+^-N plus NO_3_^−^-N over the course of field incubation is defined as total N mineralization^73^.

MBC and MBN were determined by the chloroform fumigation-extraction method using 0.5 M K_2_SO_4_ as an extractant^74^. The organic-C of extract was estimated by oxidation with potassium dichromate. The difference in the organic-C content between the fumigated and unfumigated extracts was converted to MBC by dividing with a conversion factor of 0.45^75^. The MBN was estimated by micro-Kjeldahl digestion procedure from the extracts. The difference in N content between the fumigated and unfumigated extracts was converted to MBN by dividing with a conversion factor of 0.54^76^.

For the analysis of soil base cation and non-base cations, dried soil samples in triplicate were homogenized by grinding to fine powder followed by digestion in di-acid (HNO_3_ and HClO_4_ in 9:4 ratio) solution^77^. The contents of soil Na^+^, K^+^, Al^3+^ and Fe^3+^ were determined with Atomic Absorption Spectrophotometer; AAS (Analyst-800, PerkinElmer Inc., Norwalk, CT, USA). For all the metals; blank and standards (Sisco Research Laboratories Pvt. Ltd., India) were run after every five samples to check the accuracy and precision of the results (within 2% of the certified value).

The analyses of litter decay constant, C/N ratio, pH and MBC/MBN ratio were used for explaining the patterns of soil-N pools and dynamics to the N depositions. Similarly, base and non-base cations were used. We used these variables as explanatory variables because under the scenario of N deposition these variables are supposed to be interlinked with the soil-N pools and dynamics.

For understanding the effects of N deposition on the response variables, the effect sizes (response ratio; RR) were computed following the equation of Hedges et al., (1999)^78^. For a given variable; the RR was estimated as the ratio of its value in the N treatment group (*X*_*t*_) to that in the control group (*X*_*c*_). It was transformed in log scale to improve its statistical behaviour, hence the ln *RR* = ln (*X*_*t*_/*X*_*c*_) or ln *X*_*t* _− ln *X*_*c*_ equation was used. ln *RR* was assumed to follow a normal distribution^79,80^. The optimum amount of N deposition (before the negative feedback) required for the maximum beneficial responses of the selected variables of soil-N status and dynamics were computed based on the best-fitted regression equations between the N-levels and corresponding parameters of soil-N status and dynamics.

### Statistical analyses

Repeated measures analysis of variance (RANOVA) procedure selecting general linear model (GLM) option in SPSS package^81^ was used to notice the effects of year, month, and season on the parameters of soil-N status and dynamics. In these analyses, year and season were used as within-subject variable and N-levels as between-subject factor. Tukey’s HSD test was used to determine the significance of differences in the values of these variables between different treatment pairs. Pearson correlation coefficient was established between the different response variables with the help of the SPSS package^81^. The MBN and N mineralization parameters were linearly regressed with the soil-pH, TN, C/N ratio and decay constant opting linear regression option in SPSS software^81^. A path analysis was constructed by using AMOS 16.0 software^82^, which executes the “structural equation modelling/analysis of covariance structures/causal modelling”. It was based on the linear correlation analysis and represented graphically to visualize the direct and indirect interactions among the predictors and the dependent variables^83^. In the path analysis; MBC/MBN ratio, N mineralization, inorganic-N and leaching of mineral-N were defined as dependent variables. The N-levels, soil-C:N, -pH and litter decay constant were defined as predictor variables.

## Results

### Soil pH

**Figure 2:**
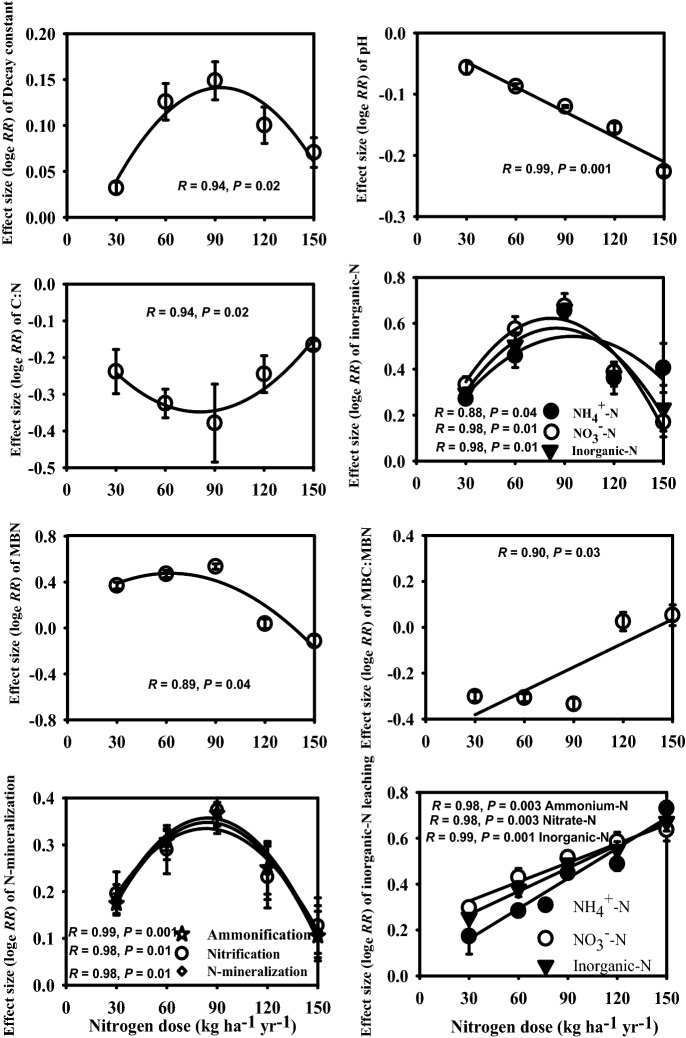
Effect size responses of soil pH, decay constant, ratios of microbial biomass carbon (MBC) to the microbial biomass nitrogen (MBN) and carbon (C) to nitrogen (N) and various soil N pools and fluxes to the different levels of experimental N input in Tropical grassland, Banaras Hindu University, Varanasi, India. Error bars represent the standard deviation. *R* = Pearson correlation coefficient, *P* = level of significance.

Season, year, N-levels and their interactions caused significant variations in the soil-pH (Table 1). The soil-pH decreased with the progress of the experimental duration and N-levels. Effect size analysis also indicated a consistent decline of soil-pH due to increasing amount of N deposition (Fig. 2).

**Table 1:** Summary of repeated measures analysis of variance (*F-*value and degree of freedom; *df*) indicating the effects of year (Y), season (S), nitrogen (N) levels and their interactions on the soil N pools and fluxes during three-year of N manipulated study in a Tropical grassland located on the campus of Banaras Hindu University, Varanasi, India.

Soil variables	Year (Y)	Season (S)	N-levels (N)	Y × S	S × N	Y × N	Y × S × N
	*F*_1,66_	*F*_1,198_	*F*_5,198_	*F*_2,198_	*F*_5,198_	*F*_5,66_	*F*_10,198_
Soil-pH	905***	6,534***	1,017***	38.76***	69.36***	37.38***	23.08***
MBN	28.42***	966***	89.40***	0.78^NS^	15.26***	1.54^NS^	3.67**
Ammonification	2,394***	17,392***	24.80***	486***	34.91***	66.93***	7.62***
Nitrification	1556***	8,144***	16.98***	151***	18.00***	37.82***	5.89***
N-mineralization	2,854***	14,878***	21.20***	351***	29.84***	72.25***	5.84***
NH_4_^+^-N	2,924***	6,611***	137***	98.61***	56.50***	107***	15.61***
NO_3_^−^-N	1744***	15,327***	236***	25.16***	437***	8.00***	69.35***
Inorganic-N	3,392***	17,150***	175***	131***	169***	58.88***	8.71***
Total-N	21.48***	1,469***	33.56***	14.34***	48.72***	1.75^NS^	3.17**
NH_4_^+^-N leaching	680***	11,099***	74.56***	164***	80.68***	11.85***	2.75*
NO_3_^−^-N leaching	5,991***	54,839***	222***	959***	325***	141***	36.74***
Inorganic-N leaching	2,861***	27,621***	136***	449***	166***	51.95***	10.90***

*MBN* microbial biomass nitrogen. *P ≤ 0.01; **P ≤ 0.001; ***P ≤ 0.0001 and ^NS^ insignificant.

### Concentrations of soil base and non-base cations

**Figure 3:**
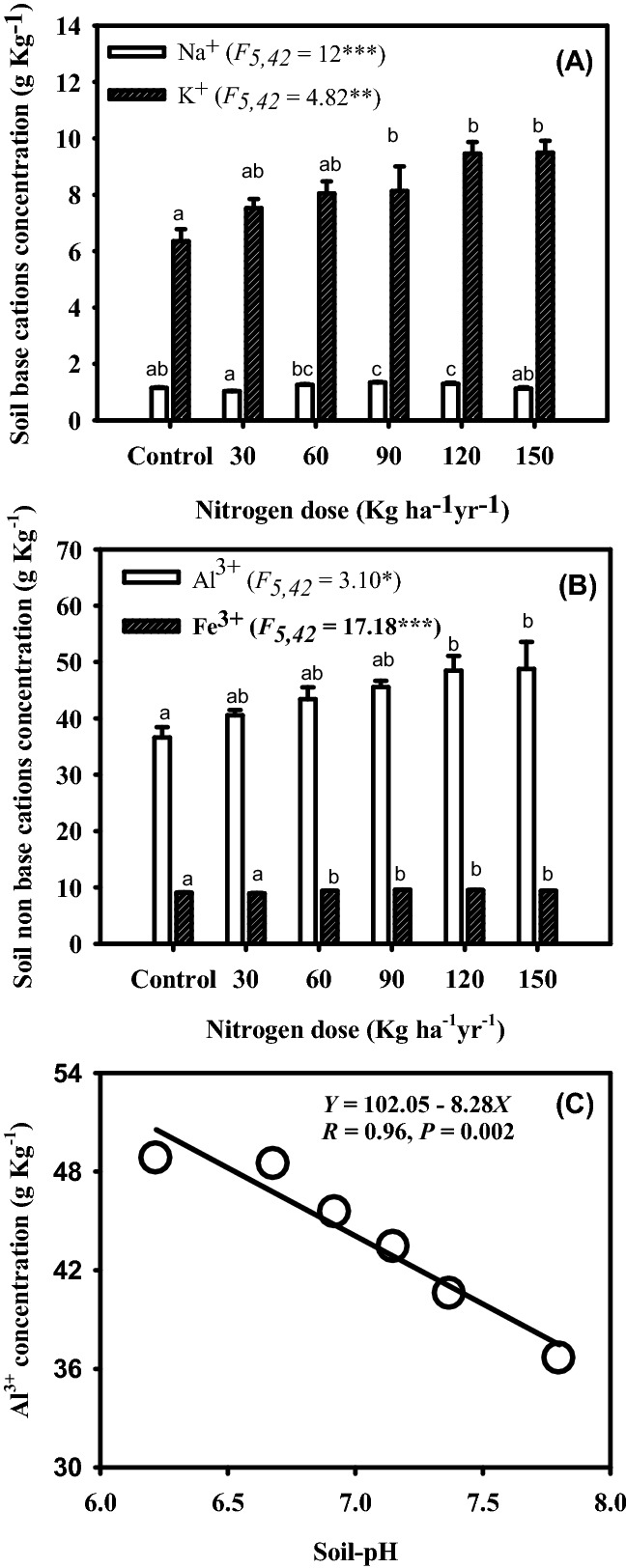
Variations in the soil base (**A**) and non-base (**B**) cations due to different levels of N inputs and relationship between concentration of Al^3+^ and soil pH (**C**) during 3-year of N manipulative study in Tropical grassland on the campus of Banaras Hindu University, Varanasi, India. The bars within a diagram affixed with different letters are significantly different from each other at *P* < 0.05.

Across the N-levels, the concentrations (g Kg^−1^ of soil) of Na^+^ and K^+^ soil base cations ranged from 1.03 to 1.34 and 6.37–7.52, respectively. The concentrations (g Kg^−1^ of soil) of Al^3+^ and Fe^3+^ (non-base cations) varied from 36.63 to 48.79 and 8.95–9.59, respectively (Table 2). The values were greater in N treated plots compared to control plots. The K^+^ (*R* = 0.97, *P*  ≤ 0.002) and Al^3+^ (*R* = 0.98, *P*  ≤ 0.001) positively responded to the N-levels (Fig. 3). Interestingly, the Al^3+^ was linearly and negatively related with the soil-pH (Fig. 3).

**Table 2:** Yearly, seasonally and nitrogen treatment-wise variations in the soil-N pools and fluxes and soil pH during 3-years of N-manipulative study in a Tropical grassland, Banaras Hindu University, Varanasi, India.

Variables	Year			Season				N-levels				
	1st	2nd	3rd	Rainy	Winter	Summer	Control	30 N	60 N	90 N	120 N	150 N
Soil-pH	7.12^c^ (0.06)	7.04^b^ (0.06)	6.92^a^ (0.06)	6.78^a^ (0.04)	7.03^b^ (0.04)	7.26^c^ (0.03)	7.80^f^ (0.02)	7.37^e^ (0.03)	7.15^d^ (0.02)	6.92^c^ (0.02)	6.68^b^ (0.03)	6.22^a^ (0.02)
MBN	66.89^b^ (6.85)	68.8^b^ (8.90)	63.42^a^ (6.36)	55.37^a^ (7.21)	65.59^b^ (6.33)	78.16^c^ (8.78)	51.76^b^ (0.85)	75.01^c^ (3.00)	83.01^d^ (3.63)	88.51^e^ (3.12)	53.74^b^ (1.47)	46.22^a^ (1.67)
Ammonification	13.79 ^b^ (0.32)	12.54^b^ (0.30)	11.36^a^ (0.27)	18.88^c^ (0.26)	9.70 ^b^ (0.19)	8.76^a^ (0.17)	10.17^a^ (0.35)	12.12^abc^ (0.51)	13.87^cd^ (0.66)	14.70^d^ (0.70)	13.16^bcd^ (1.16)	11.36^ab^ (0.87)
Nitrification	9.52^b^ (0.25)	8.80^b^ (0.23)	7.59^a^ (0.19)	13.34^c^ (0.20)	6.80^b^ (0.15)	5.90 ^a^ (0.12)	6.98 ^a^ (0.23)	8.51^c^ (0.50)	9.35^c^ (0.58)	10.18^d^ (0.71)	8.86^cd^ (0.81)	7.97^ab^ (0.66)
N-Mineralization	23.31^b^ (0.56)	21.34^b^ (0.52)	18.95 ^a^ (0.46)	32.22^c^ (0.45)	16.50^b^ (0.33)	14.66^a^ (0.27)	17.15^a^ (0.58)	20.63^abc^ (1.00)	23.22^cd^ (1.21)	24.88^d^ (1.41)	22.02^bcd^ (1.97)	19.33^ab^ (1.53)
NH_4_^+^-N	7.11^c^ (0.19)	6.47^b^ (0.13)	5.27^a^ (0.15)	4.22^a^ (0.10)	6.39^b^ (0.12)	8.24^c^ (0.16)	4.61^a^ (0.17)	5.91^c^ (0.20)	6.97^d^ (0.20)	8.59^e^ (0.26)	6.16^cd^ (0.21)	5.45^b^ (0.19)
NO_3_^—^N	4.34^c^ (0.11)	3.86^b^ (0.12)	3.54^a^ (0.10)	2.60 ^a^ (0.05)	3.77^b^ (0.06)	5.36^c^ (0.11)	2.66^a^ (0.09)	3.68^c^ (0.08)	4.73^e^ (0.12)	5.20^f^ (0.19)	3.99^d^ (0.16)	3.20^b^ (0.12)
Inorganic-N	11.44^c^ (0.29)	10.33^b^ (0.23)	8.81 ^a^ (0.24)	6.83 ^a^ (0.13)	10.16^b^ (0.18)	13.60^c^ (0.24)	7.28^a^ (0.25)	9.59^c^ (0.28)	11.70^e^ (0.31)	13.79^f^ (0.43)	10.15^d^ (0.34)	8.65^b^ (0.27)
Total-N	1.65^a^ (0.17)	1.90^b^ (0.21)	1.79^b^ (0.20)	2.49^c^ (0.31)	1.70^b^ (0.20)	1.15^a^ (0.07)	1.23^a^ (0.06)	1.60^bc^ (0.09)	2.23^d^ (0.10)	2.44^d^ (0.13)	1.74^c^ (0.07)	1.43^ab^ (0.03)
NH_4_^+^-N Leaching	1.65^a^ (0.10)	2.14^b^ (0.14)	2.50^c^ (0.15)	4.42^c^ (0.09)	1.61^b^ (0.06)	0.26^a^ (0.01)	1.42^a^ (0.14)	1.73^b^ (0.16)	1.90^c^ (0.16)	2.23^d^ (0.18)	2.33^e^ (0.19)	2.97^f^ (0.24)
NO_3_^—^N Leaching	2.85^a^ (0.14)	3.83^b^ (0.19)	4.54^c^ (0.23)	6.99^c^ (0.14)	3.58^b^ (0.08)	0.64^a^ (0.02)	2.41^a^ (0.18)	3.23^b^ (0.23)	3.74^c^ (0.28)	4.04^d^ (0.28)	4.38^e^ (0.31)	4.62^f^ (0.33)
Inorganic-N Leaching	4.49^a^ (0.24)	5.97^b^ (0.32)	7.04^c^ (0.38)	11.41^c^ (0.22)	5.19^b^ (0.13)	0.90^a^ (0.02)	3.83^a^ (0.32)	4.96^b^ (0.39)	5.64^c^ (0.43)	6.27^d^ (0.46)	6.71^e^ (0.50)	7.59^f^ (0.56)

The rates of ammonification, nitrification and N mineralization are in µg g^−1^ month^−1^. The values of soil total-N are in g kg^−1^ of soil. The values of inorganic-N (NH_4_^+^-N, NO_3_^−^-N), N-leaching, microbial biomass nitrogen (MBN) are in µg g^−1^. The values in parentheses are ± 1*SE*. The means within a column superscript with different letters within a predictor variable are significantly different from each other at *P* ≤ 0.05.

### Soil microbial biomass nitrogen (MBN)

**Figure 4:**
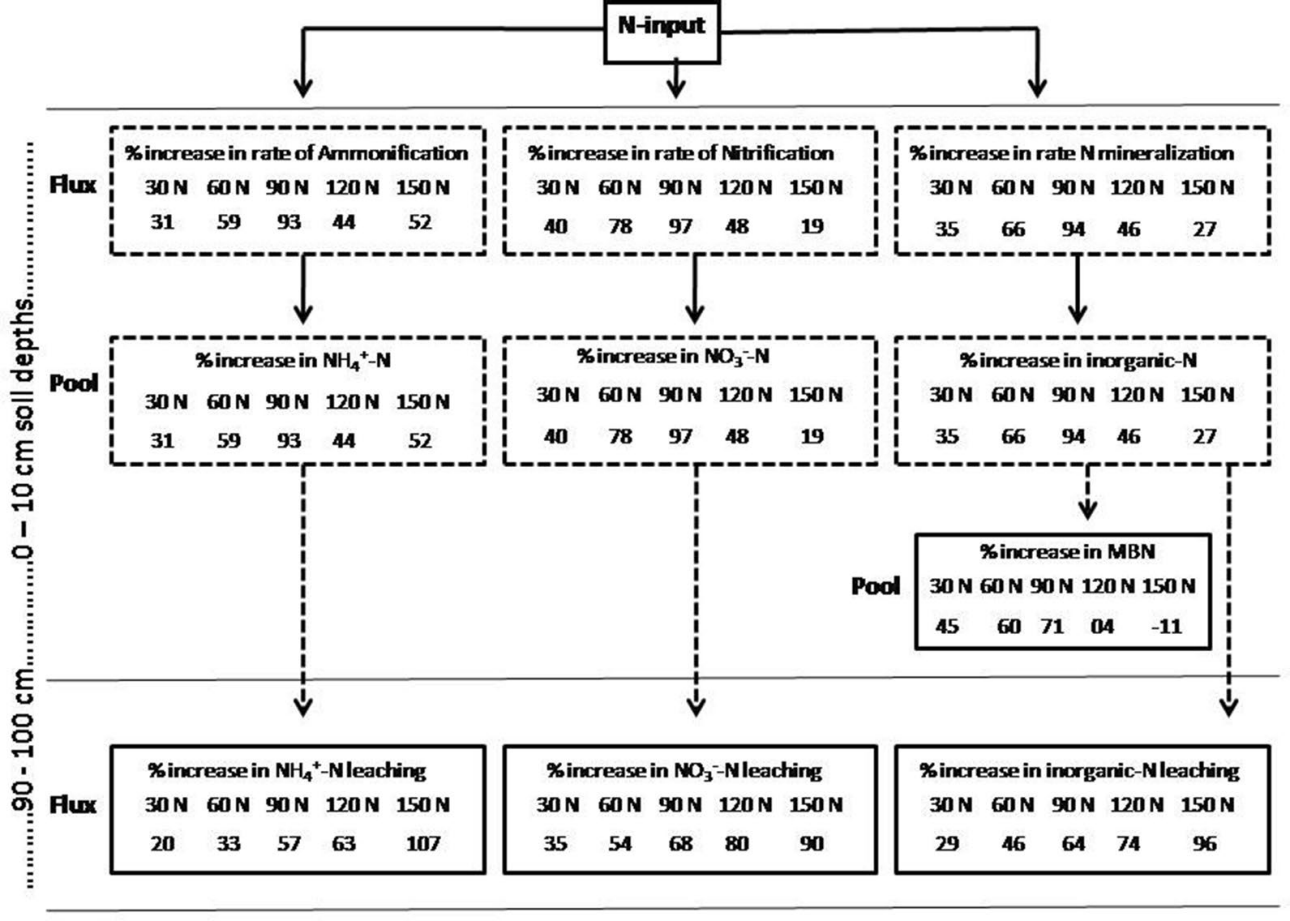
A conceptual framework for the percentage change in soil N pool and fluxes at 0 to 10 cm soil depth and proportional change in the leaching of NH_4_^+^-N, NO_3_^−^-N and inorganic-N to the three-year of N manipulative study in Tropical grassland, Banaras Hindu University, Varanasi, India.

Across the N-levels, MBN (µg g^−1^) varied from 415; N_150_ level to 545; N_90_ level (Table 2). RANOVA showed significant effects of year, season, N-levels, season × year, season × N-levels, year × N-levels and season × year × N-levels on the MBN (Table 1). Yearly, the MBN was maximum in second-year of the experiment and minimum in the third-year of the experiment (Table 2). Tukey’s HSD test showed significant differences in the values of the MBN among the different year-pairs (Table 2). Season-wise, it was minimum in rainy and maximum in the dry season (Table 2). The Tukey’s test yielded a significant difference in the MBN between rainy and winter, between winter and summer and between summer and rainy seasons (Table 2). Response size of the MBN to the N-levels was humped-shape (Fig. 2). Compared to control, the percent change in the MBN for each N-level varied between -11; N_150_ level and 21; N_90_ level (Fig. 4).

### Rate of N-mineralization

Across the N-levels; ammonification, nitrification and total N-mineralization ranged from 10.17 to 14.70, 6.98–10.18, and 17.15–22.02, respectively (Table 2). The values of these parameters were low in N_0_ and high in N_90_ (Table 2). Year wise, the ammonification (µg g^−1^ month^−1^), nitrification (µg g^−1^ month^−1^) and total N-mineralization (µg g^−1^ month^−1^) varied between 11.36 and 13.79, 7.59 and 9.52, and 18.95 and 23.31, respectively. The values were minimum for the third-year of the experiment and maximum for the first-year of the experiment. Minimum values were observed in the dry season and maximum in the rainy season (Table 2). Results showed differences in the values of total N-mineralization as well as its components due to differences in the year, season, N-levels and their possible interactions (Table 1). The Tukey’s test suggested major variations in the values of the third-year N-mineralization with those of the first and second years. Similarly, these parameters varied between different season-pairs (Table 2). The response effect sizes of these parameters to the N-levels were quadratic (Fig. 2). Compared to the N_0_, the percent changes in ammonification, nitrification and total N-mineralization for each N-level varied from 31 to 93, 19–97 and 27–94. Also, these parameters showed quadratic responses to the N-levels (Fig. 4). The ammonification, nitrification and net N-mineralization were positively related with the decay constant (*R* = 0.37–0.45, *P* ≤ 0.001, *n* = 72), negatively with C/N (− *R* = 0.54–60, *P* ≤ 0.001, *n* = 72) and MBC/MBN ratios (− *R* = 0.58–0.59, *P* ≤ 0.001, *n* = 72) and quadratically with the soil-pH (*R* = 0.46–0.54, *P* ≤ 0.001, *n* = 72).

**Figure 5:**
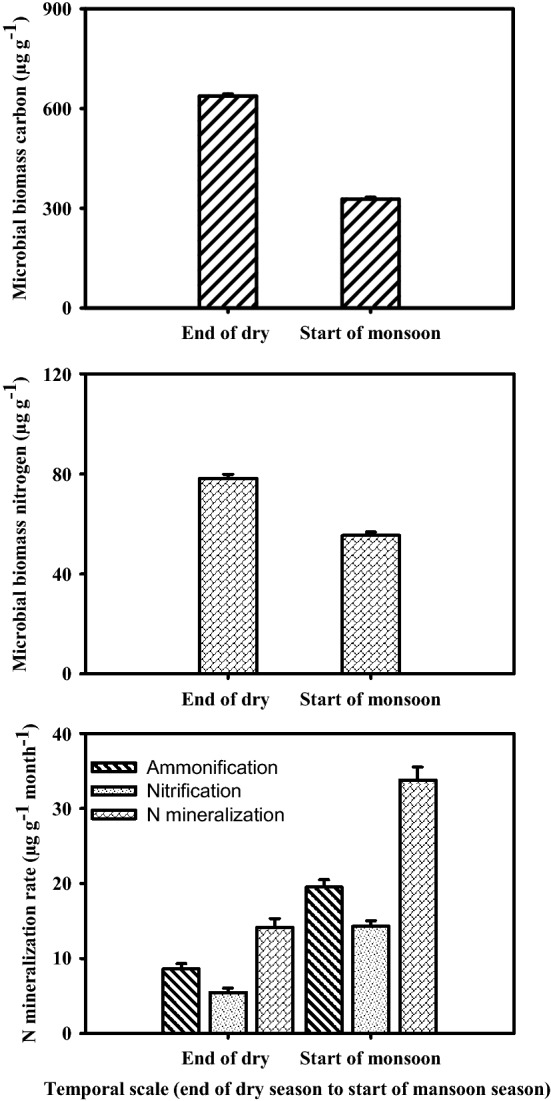
Comparison of microbial biomass carbon (MBC), nitrogen (MBN) and parameters of N mineralization (µg g^−1^ month^−1^) from the end of dry season to the start of rainy season during a 3-year of N manipulative study in Tropical grassland, Banaras Hindu University, Varanasi, India.

The ammonification, nitrification and total N-mineralization promptly increased from the end of the dry season to the onset of the rainy season (Fig. 5). Such pattern indicated a quick response of N mineralization to the first-rain event after a period of dry season.

### Mineralized N (NH_4_^+^-N, NO_3_^−^-N, inorganic-N)

Yearly*,* the amount of NH_4_^+^-N, NO_3_^−^-N, inorganic-N (µg g^−1^) varied from 5.27 to 7.11, 3.54–4.31 and 8.81–11.44, respectively (Table 2). In the same order, the seasonal values of these parameters ranged from 4.22 to 8.24, 2.60–5.36 and 6.83–13.60. Across the N-levels, their corresponding values ranged between 4.61 and 8.59, 2.66 and 5.20, and 7.28 and 13.79 (Table 2). The values of these variables were minimum in the third-year of the experiment and maximum in the first-year of the experiment. These results suggested reduction of available-N due to longer period of N deposition. Season-wise, the values of available-N were minimum in the rainy season and maximum in the summer season. Similarly, N level-wise, the values were minimum in the N_0_ and maximum in the N_90_ levels (Table 2).

**Figure 6:**
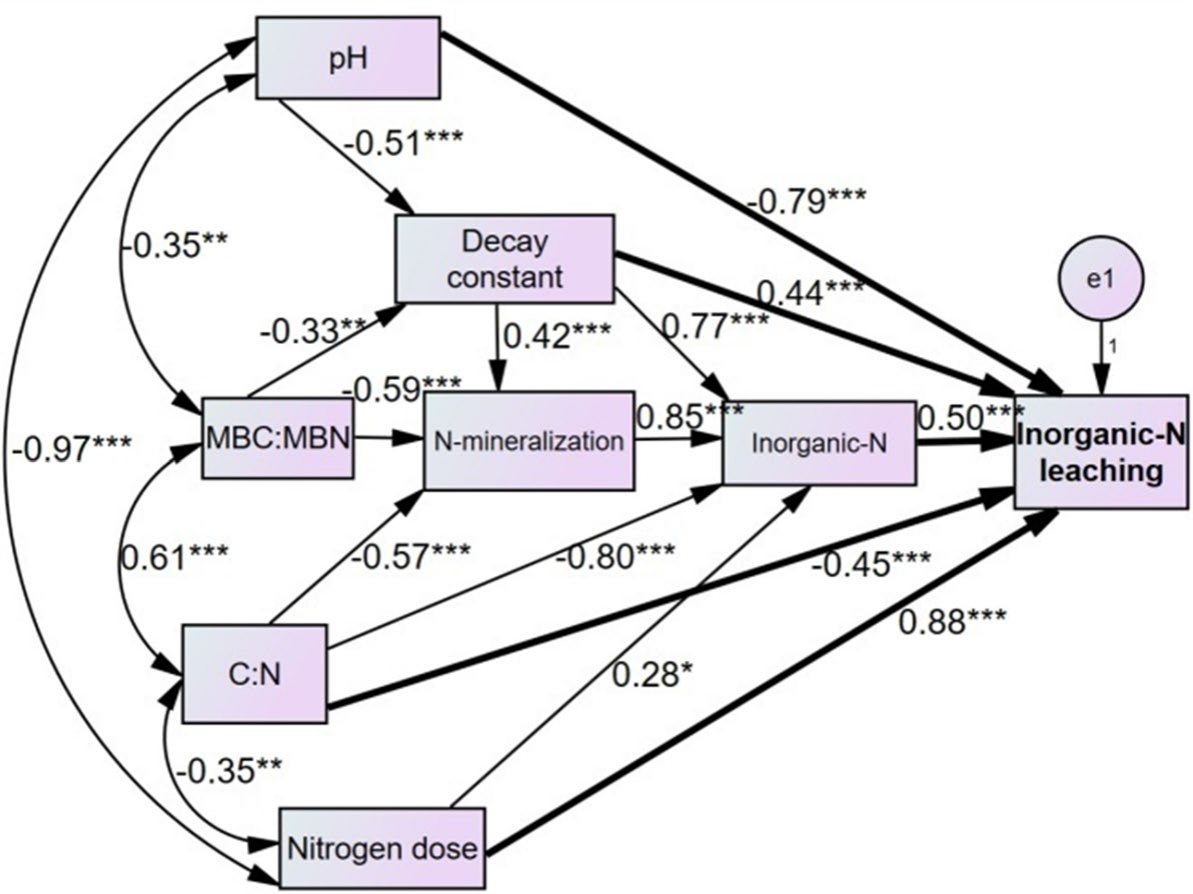
Path analysis indicating the direct (bold arrows) and indirect (thin arrows) effects of soil variables on the soil-N pools and fluxes during 3-year of N manipulative study in Tropical grassland, Banaras Hindu University, Varanasi, India. The path coefficients are standardized regression coefficients. The values associated with arrows represent standardized path coefficients. *, ** and *** represent significance levels of standardized path coefficients at *P* < 0.05, *P* < 0.01 and *P* < 0.001, respectively.

RANOVA showed considerable variations in the NH_4_^+^-N, NO_3_^−^-N and inorganic-N due to year, season, N-levels and their interactions (Table 1). Moreover, the mineralized N substantially varied between the years and between the seasons (Table 2). The effect size responses of the mineralized-N to the N-levels were positive and humped-shape (Fig. 2). The results showed synchronization of NH_4_^+^-N, NO_3_^−^-N and inorganic-N with those of the ammonification, nitrification and total N-mineralization. Thus, it seems that the available-N was controlled by the rates of N-mineralization as also suggested by path analysis (Fig. 6). Interestingly, similar to the N-mineralization attributes; the NH_4_^+^-N, NO_3_^−^-N and inorganic-N were positively related with the decay constant (*R* = 0.74–0.77, *P* ≤ 0.001, *n* = 72), negatively with the C/N (− *R* = 0.76–80, *P* ≤ 0.001, *n* = 72) and MBC/MBN ratios (− *R* = 0.52–0.73, *P*  ≤ 0.001, *n* = 72) and quadratically with the soil-pH (*R* = 0.68–0.74, *P* ≤ 0.001, *n* = 72).

### Leaching of NH_4_^+^-N, NO_3_^−^-N and inorganic-N

RANOVA yielded remarkable differences in the leaching of NH_4_^+^-N, NO_3_^−^-N and inorganic-N due to year, season, N-levels and their interactions (Table 1). Individually, these attributes were one and half times greater in the third-year of the experiment than the first-year of the experiment, 11–17 times greater in the rainy season than the dry season and consistently increased with the N-levels (Table 2). Interestingly, the values were two-fold greater in the N_150_ than the N_0_ (Table 2). Also, the effect size analysis indicated increasing pattern of N leaching along the increasing rate of N depositions (Fig. 2). Compared to control, N deposition favoured the leaching of NH_4_^+^-N by 20–107%, NO_3_^−^-N by 33–95% and inorganic-N by 29–97%. Overall, on average N deposition enhanced leaching of NH_4_^+^-N by 56% and NO_3_^−^-N by 65% (Fig. 4).

Pearson correlation analysis indicated that the leaching of NH_4_^+^-N, NO_3_^−^-N and inorganic-N were positively related with TN, decay constant and rates of N-mineralization (Table 3). In contrast to these relations, the N leaching parameters were negatively related with those of soil-pH, MBN, C/N ratio, MBC/MBN ratio, NH_4_^+^-N, NO_3_^−^-N and inorganic-N of the 0–10 cm soil depth (Table 3).

**Table 3:** Correlation matrix indicating the relationships of nitrogen (N) leaching with the N-levels, soil-pH, decay constant, microbial biomass (MBN), microbial biomass carbon (MBC): MBN, rates of N mineralization, inorganic N, total N and carbon : nitrogen (C:N) during the three-year of N manipulative study in Tropical grassland located on the campus of Banaras Hindu University, Varanasi, India.

Variables	NH_4_^+^-N leaching	NO_3_^−^-N leaching	Inorganic-N leaching
N-levels	0.25**	0.25**	0.25**
pH	− 0.52**	− 0.55**	− 0.55**
Decay constant (k)	0.92***	0.94***	0.94***
MBN	− 0.33**	− 0.33**	− 0.33**
MBC:MBN	− 0.40**	− 0.42**	− 0.42**
Ammonification	0.75***	0.69***	0.72***
Nitrification	0.75***	0.69***	0.72***
N-mineralization	0.75***	0.69***	0.72***
NH_4_^+^-N	− 0.54**	− 0.56**	− 0.56**
NO_3_^−^-N	− 0.60***	− 0.61***	− 0.62***
Inorganic-N	− 0.59**	− 0.61***	− 0.61***
Total-N	0.64***	0.66***	0.66***
C:N	− 0.40**	− 0.44**	− 0.43**

Values are Pearson correlation coefficients (*R*) ^NS^insignificant, *significant at *p* ≤ 0.05; **significant at *p* ≤ 0.01; ***significant at *p* ≤ 0.001.

Finally; path analysis was performed to identify the direct and indirect effects of soil variables on the parameters of inorganic-N leaching. Results showed that the N-levels, soil-pH, decay constant, N-mineralization and C/N ratio caused direct effects on the leaching of inorganic-N. As a main result; the rate of N deposition and soil-pH mediated by it firmly determined the leaching of inorganic-N (Fig. 6).

## Discussion

### Soil acidification, soil base and non-base cations

In the present study, N induced reduction of soil-pH along increasing rate of N deposition could be due to increase in Al^3+^ concentration. It is evident because of negative relationship of Al^3+^ concentration with soil-pH. Release of carbonic acids during litter decomposition^84^ and H^+^ into the soil solutions during nitrification^85^ could be probable explanation for reduction of soil-pH owing to N deposition. Also, it is known that breakdown of urea fertilizer (similar to other studies^65,66^, we also used urea fertilizer to simulate N deposition) in presence of soil moisture and uptake of NH_4_^+^ through the plant roots decrease the soil-pH^21,31,84^. In other studies differential buffering and mobilization capacities of base and non-base cations are suggested for soil acidification because of N input^57^. Reports indicated that reduction of base cations (Na^+^ and K^+^) present in soil system usually delays acid-buffering capacity of the soil^57,86^. Once, these base cations are exhausted, the non-base cations (Al^3+^ and Fe^3+^) mobilize and buffer during the N-induced soil acidification^57,86^. Similar to present study, other studies also reported reduction of soil-pH due to N deposition^21,23,55,66^.

### Microbial biomass-N

Low MBN in the rainy season and high in the dry season suggested seasonality in the MBN of the tropical grassland^29,87,88^^.^ Significantly positive effect sizes of MBN from low to moderate N-levels (N_30_-N_90_), and negative from moderate to high levels of applied N (N_120_-N_150_), decline in the third-year of the experiment and humped-shape response across the applied N-levels (by pooling entire data) favoured the assumption that moderate level of N deposition favours maximum MBN while continuous and sufficiently high amount of N depositions retard the conservation of soil-N in microbial biomass^89,90^. Noticeably, regression analysis revealed 63 kg N ha^−1^ year^−1^ as an optimum rate of N deposition for maximum accumulation of N in the microbes of tropical grassland. Using 82 published field studies (considering only highest N application rates), Treseder^91^ suggested reduction of microbial biomass due to N additions. However, he excluded the data of microcosm or greenhouse-based experiments as well as organic N or urea, or N with phosphorus added data. A recent analysis of Camenzind et al.^52^, from tropical forest showed inconsistent (no relation to N in lowland forest and positive in montane forests) responses of microbes to the N deposition. In the present study, occurrence of positive relation between MBN and soil inorganic-N pool, and maximum MBN at 63 kg ha^−1^ year^−1^ N deposition rate improved our understanding that the N-deposition dependent MBN of the tropical grassland may act as a source for the availability of mineral-N in the soil system.

### N mineralization

The occurrence of maximum total N-mineralization in the rainy season and minimum in the dry season suggested a flush of N-mineralization at the start of the rainy season. It may be because of drying and rewetting effects of the dry tropical soils^21,29,92,93^. Rapid variation in soil water potential because of drying and rewetting possibly exhibited osmotic shocks to the microbes; consequently, there could have microbial cell lysis and release of cell solutes^94^. The labile-N substrate quickly mineralized through the activities of the remaining microbes; hence, a pulse of N-mineralization was likely^95^. It might be expected that the microbes have stored a higher amount of N during the dry season and as they receive a rain event their activities get accelerated, consequently, the start of the rainy season yielded a greater mineralised-N^29,96^.

The study yielded humped-shape curves for the effect sizes of N-mineralization parameters to the N-levels. These curves suggested that the N-mineralization was low at low levels of N depositions (N_30_–N_90_), whereas increased to a maximum at a moderate level (N_90_) then decreased towards the higher rate of N deposition (N_90_–N_150_). It could be explained by changes in composition and activities of soil microbes,’ soil-pH and rate of organic matter decomposition in response to N depositions. These patterns are evident due to positive relations of decay constant, negative relations of C/N and MBC/MBN ratios and quadratic relations of soil-pH with those of ammonification, nitrification and total N-mineralization. The path analysis also revealed such mechanisms for the controlling the N-mineralization in the present experiment.

Since the N-mineralization parameters quadratically responded to the N deposition and soil-pH and 90 kg N ha^−1^ year^−1^ deposition rate yielded 6.98 soil-pH for maximum N-mineralization, therefore, 90 kg N ha^−1^ year^−1^ deposition is thought to be an optimum amount of N for favourable soil-pH that had supported maximum soil N-transformation from organic residues to the mineral-N in the tropical grassland. Similar to the present findings, other temperate studies also suggested moderate level of N deposition for greater soil N-mineralization mediated by microbial communities and their performances^36–38,97–99^. The poor quality of decomposing materials; high C/N ratio^43,84,100^, poor growth and activities of oligotrophic decomposers; high MBC/MBN ratio^43,101–103^ and conditions during the decomposition; low soil-pH and high Al^3+^ toxicity^104^, could be major constrains for the transformation of organic residues into the mineralized-N.

### Soil mineral-N pool

Similar to present study other temperate studies also suggested positive relationships between soil mineral-N status (NH_4_^+^-N, NO_3_^−^-N) and the rate of N-mineralization under the N-deposition scenario^31,84,105^. The synchronisation of NH_4_^+^-N and NO_3_^−^-N with those of ammonification and nitrification in relation to the N deposition also highlighted that NH_4_^+^-N and NO_3_^−^-N are low at the low level of N deposition, became maximum at a medium level of N deposition (90 kg N) and decline progressively towards higher N deposition levels. The study inferred that at the low level of N deposition; probably the soil-N was not sufficient for the activities of ammonifiers and nitrifiers to release the NH_4_^+^-N and NO_3_^−^-N. As soon as amount of N deposition was increased, more ammonifiers and nitrifiers get activated for ammonification and nitrification^23^, at adequately high level of N deposition, probably there was loss of additional N via volatilization of ammonium^106^, denitrification of nitrate^106–108^ and also, possibly consumed by the nitrophilc plants for increasing their biomass^23,109–111^.

Further, study believes that whatever N is present in the control plot maybe because of biological N_2_-fixation as well as from the release of N through the microbial decomposition of litters. The N deposition at the rate of 90 kg ha^−1^ year^−1^ reduces the N_2_-fixation for a short time and later on; N_2_-fixation is increased because of an increased microbial population^23^. Beyond this limit of N deposition; denitrification (if any); appears to be insignificant; therefore, the loss of extra N may be through volatilization^106^ and denitrification^112^, however, at a slow rate. The N deposition at the rate of 150 kg ha^−1^ year^−1^ entirely reduces the growth of N_2_-fixing microbes and N_2_-fixation as well as activities and the growth of denitrifying microbes^106,112^. Additionally, a substantial amount of NH_4_^+^-N and NO_3_^−^-N may be taken by the nitrophilic species^23,109,110^. In this situation; the quantity of ammonia formation appeared to be reasonably high because of the increased quantity of substrate^106,113,114^ and most of this ammonia is being transformed into the NO_3_^−^-N by nitrifies, whereas remaining ammonia is being volatilized^106^.

### Mineral-N leaching

Significantly positive linear relationships of NH_4_^+^-N, NO_3_^−^-N and total inorganic-N to the N-levels as well as yearly increased amount of these variables in the 90–100 cm soil depth suggested that the increased amount of N deposition probably exceeded beyond the needs of microbes and plants. Therefore, extra N could have percolated in the 90–100 cm soil depth^115–117^. In contrast to 0–10 cm soil depth; the amounts of NH_4_^+^-N, NO_3_^−^-N and inorganic-N at 90–100 cm soil depths were maximum in the rainy season and minimum in the dry season. The emergence of such patterns may be due to the maximum uptake of mineral-N by the plants for their vigorous growth in the rainy season. At the same time, the increased precipitation and slightly warmer condition during rainy season probably increased the microbial activities and the rates of decomposition which in turn could have increased the rate of N-mineralization (because of positive relationship between decay constant and N-mineralization). Thus, the remaining inorganic-N beyond the demands of plants and microbes possibly resulted into the leaching of inorganic-N through the water^2^. Probably, it could be a reason for high leaching in rainy and low in the dry season.

The percentage change of NO_3_^−^-N leaching is approximately one and a half times greater than the NH_4_^+^-N. It may be because of greater nitrification and accumulation of NO_3_^−^-N (as evident by positive relationship between nitrification and NO_3_^−^-N leaching) which were assumed to be over and above the requirements of the plants and nitrifying microbes and less competition between them for NO_3_^−^-N^118,119^. Further, higher NO_3_^−^-N leaching rates than that of NH_4_^+^-N may be expected because the latter is a preferred form of inorganic N for the biota due to low energetic cost during biological assimilation. Thus, it appears that the high NO_3_^−^-N leaching could be a dominant form of N-leaching because of biological assimilation controlled NO_3_^−^-N saturation^120,121^. Also, the possible mechanisms behind it may be explained by the differences in the charges of NH_4_^+^ and NO_3_^−^. For example, the NH_4_^+^ is positively charged and it binds with the negatively charged clay particles while the negatively charged NO_3_^−^ freely moves with the water molecule until the exchange of anions within the soil is completed^122^. Thus, the study supported the view that the excess N deposition increases the NO_3_^−^-N leaching^2,58^ in the tropical grassland. Its proper management is warranted; otherwise the excess N deposition may cause soil acidification, leaching of N^123^ and ultimately participate in the warming of the globe through production of nitrous oxide from soil via nitrification and denitrification by different aerobic and anaerobic microbes^124^. Overall, the current holistic study revealed that the N deposition below 90 kg ha^−1^ year^−1^ could be a substantial limit for the healthy soil-N fertility and its transformation in the tropical grassland.

## Conclusions

The continuous, as well as an incremental amount of N-levels decreased the soil-pH and increased the Al^3+^ concentration within the soil system and changes in these soil variables governed the decomposition of organic materials and N-transformation. Also, the N deposition dependent soil-pH, decay constant and N-mineralization guided the leaching pattern of mineral-N. The N deposition below 90 kg ha^−1^ year^−1^ seems to be an optimum limit for the maximum soil-N status and dynamics. The N deposition beyond this limit caused negative feedback to the soil-N fertility and its dynamics. Hence, this holistic approach suggested that the N deposition should not go beyond 90 kg ha^−1^ year^−1^ and it should be managed by implementing into a policy for sustainable functioning of the tropical or similar grasslands.
